# Investigating risk-taking and executive functioning as predictors of driving performances and habits: a large-scale population study with on-road evaluation

**DOI:** 10.3389/fpsyg.2023.1252164

**Published:** 2023-11-27

**Authors:** Pierre Le Denmat, Fanny Grisetto, Yvonne N. Delevoye-Turrell, Quentin Vantrepotte, Tanguy Davin, Andreea Dinca, Isabell Desenclos-El Ghoulti, Clémence Roger

**Affiliations:** ^1^Univ. Lille, CNRS, UMR 9193 – SCALab – Sciences Cognitives et Sciences Affectives, Lille, France; ^2^Department of Brain and Cognition, KU Leuven, Leuven, Belgium; ^3^Centre de Recherche de l’Ecole de l’Air, Salon-de-Provence, France; ^4^ECCA Conduite, Lyon, France

**Keywords:** driving behaviors, cognitive control, inhibition, adaptive mechanisms, risk-taking, strategical compensation

## Abstract

**Introduction:**

Maladaptive behavior often results from poor decision-making and by extension poor control over decisions. Since maladaptive behavior in driving, such as excessive speed, can lead to dramatic consequences, identifying its causes is of particular concern. The present study investigated how risk-taking and executive functioning are related to driving performance and habits among the general population.

**Method:**

Five hundred and eighty-nine participants completed an on-road driving session with a professional driving instructor and a self-reported checklist of difficult driving situations typically avoided. Additionally, participants completed a set of experimental tasks assessing risk-taking tendencies, reactive adaptive mechanisms, and two distinct forms of inhibition: interference control and response inhibition.

**Results:**

The results of the present study revealed several significant findings. Firstly, poor driving performance was associated with a high avoidance of challenging driving situations. Secondly, neither form of inhibition studied (interference control or response inhibition) predicted driving performance. Thirdly, while greater involvement in reactive adaptive mechanisms did not predict better on-road performance, it was associated with a reduced tendency to avoid difficult situations. Surprisingly, a higher propensity for risk-taking predicted better on-road performance.

**Discussion:**

Overall, these results underline limited links between executive functioning and driving performance while highlighting a potentially more complex relationship between risk-taking tendencies and driving. Executive functioning, however, appears to be linked to driving habits.

## 1 Introduction

When driving to an unknown destination, it is imperative to remain attentive to the directions provided by the navigation system to ensure one is still following the right itinerary. If individuals happen to notice the required change in direction too late, they are faced with a decision: either attempt a sudden course correction, potentially putting themselves at risk, or continue and adjust their route later. Many other driving scenarios require rapid decision-making regarding the most appropriate behavior to adopt: overtaking a truck with limited sight distance, making quick braking decisions to avoid obstacles, or negotiating a junction with other approaching vehicles. Driving, as a complex task of everyday life, thus engages high-level cognitive functions involved in decision-making and action control (Anstey et al., 2005), which are part of the executive functions. When these functions are weakened for diverse reasons, errors, and, by extension car crashes, have a higher probability of occurring. On a related note, personality traits, such as risk-taking tendencies, can lead individuals to make inappropriate decisions despite having fully operational cognitive functions. Considering the frequency of driving behavior and the potential for maladaptive behavior to have dramatic consequences on road safety, it is particularly important to identify the cognitive and non-cognitive factors associated with unsafe driving performance.

Executive functions can be defined as a set of high-order cognitive processes that are required to optimize performance in complex tasks, allowing individuals to adapt their behavior according to internal goals and environmental demands (Ridderinkhof et al., 2011). To date, several studies have investigated the links between these executive capacities and driving performance. These investigations seek to explain poor driving performance and the increased risk of being involved in car accidents among specific populations due to executive impairments. Decreased executive functioning has been shown to predict poorer driving performance in individuals suffering from pathologies such as multiple sclerosis, Alzheimer’s disease, attention deficit hyperactivity disorder, and persons suffering from acquired cognitive impairments (Asimakopulos et al., 2012). Additionally, numerous studies have examined the impact of executive function capacities on both healthy older drivers (Adrian et al., 2011, 2019; Anstey and Wood, 2011) and young novice drivers (Walshe et al., 2017). Overall, results varied depending on the specific driving metrics used and executive functions tested. In this set of several high-order cognitive processes, Miyake et al. (2000) indeed extracted three main components of executive functions: shifting, updating, and inhibition. These three executive functions have been specifically investigated in relation to driving performance.

The shifting function consists of switching the focus of attention between multiple sources of information, a skill frequently used while driving. However, in the case of healthy populations, the majority of studies that examined the shifting function did not establish a specific link between driving performance and shifting capacities (Mäntylä et al., 2009; Graefe and Schultheis, 2013; Guinosso et al., 2016; Pope et al., 2016). It is worth noting that a few counterexamples can be found in the literature (Adrian et al., 2011; Anstey and Wood, 2011).

The updating function, closely related to working memory, monitors and updates the information relevant to the task at hand (e.g., speed limitations while driving). Mäntylä et al. (2009) reported a positive correlation between updating capacities and driving performance. However, this result was not replicated in Graefe and Schultheis (2013) and Pope et al. (2016). Interestingly, even contradictory results have been reported in Ross et al. (2015). On the one hand, verbal working memory correlated negatively with lateral lane position variability, indicating better driving performance in individuals with better updating ability. On the other hand, individuals with better visuospatial working memory ability exhibited more instances of running yellow-light and reduced time headway (i.e., following distances), suggesting riskier driving behavior. This former result challenges the assumption that enhanced updating capacity invariability leads to safer driving.

The inhibition function plays a crucial role in enabling individuals to effectively filter out irrelevant information, withhold automatic and inappropriate responses, and interrupt ongoing actions that are not, or no longer, suited to the situation (e.g., lifting your foot from the accelerator when the light turns red). Most studies reported that poorer inhibition capacities were associated with poorer driving performances (O’Brien and Gormley, 2013; Ross et al., 2015, 2016; Hatfield et al., 2017). However, it is important to note that this pattern was not replicated in every inhibition experimental paradigm (e.g., the Go/NoGo task and the Stop Signal task), nor for every driving metric measured (e.g., mean and maximum speed, yellow-light running, lateral lane position variability, traffic offense history). Accordingly, Mäntylä et al. (2009) and Graefe and Schultheis (2013) reported no significant effect of inhibition on driving performance.

In general, studies evaluating executive functions and their impact on driving performance have yielded varied conclusions. These mixed results, depending on the specific cognitive function assessed and the driving metric used, thus suggest a more complex relationship between executive functions and driving performance. Furthermore, these results do not provide insight into the role of general executive functioning in driving. Adrian et al. (2019) in their study employed a paradigm similar to Miyake et al. (2000) to evaluate each executive component and have a global overview of the executive functioning of each participant. Each component (i.e., shifting, updating, and inhibition) was characterized as a composite variable underlying the performance in three different tasks evaluating the same component. Using structural equation modeling to assess which specific executive functions impact on-road driving performance, the authors concluded that inhibition was the only reliable predictor of driving performance. However, it is crucial to note that the inhibition composite variable considered in Adrian et al. (2019) actually included two distinct forms of inhibition: response inhibition and interference control. Inhibition is often described as a unitary concept, yet it encompasses two different constructs, namely the ability to ignore irrelevant information and the ability to stop responses in the course of planning or even execution (Rey-Mermet et al., 2017). Indeed, in their study, the three experimental tasks used to assess inhibitory capacities were the Stroop task (Stroop, 1935), the Go/NoGo task (Simson et al., 1977), and an incompatibility test (Zimmermann and Fimm, 2004). The Go/NoGo task and the Stroop task are considered to mainly measure the effectiveness of the response inhibition process. Response inhibition refers to the act of preventing or actively suppressing an engaged or automatic response, as operated by participants in both the Go/NoGo and the Stroop tasks (i.e., inhibiting erroneous dominant or automatic responses, respectively). Conversely, the incompatibility test is considered to assess interference control that implies selecting the appropriate, intention-based, action and hence inhibiting interfering information that could lead to erroneous response selection. In this task, participants encounter conflicting scenarios that require them to resist the interference caused by prominent behavior.^1^ Even though Friedman and Miyake (2004) found a relatively strong relationship between interference control and response inhibition, the existence of different forms of inhibition has been outlined in several studies as low to no correlation between different inhibition tasks were reported (Kramer et al., 1994; Grant and Dagenbach, 2000; Friedman and Miyake, 2004; Stahl et al., 2014; Rey-Mermet et al., 2017). Building on the premise that inhibition might have a key role in driving according to the results of Adrian et al. (2011), there remains a gap in understanding the respective contributions of each form of inhibition to driving performance.

In addition to investigating precisely the various inhibitory processes underlying online action control, it is interesting to examine the involvement of anticipatory action regulation mechanisms. These mechanisms aim to modulate the level of involvement of online action control before being confronted with demanding situations (Ridderinkhof et al., 2011). According to Ridderinkhof et al. (2011), these mechanisms can be classified as either reactive or prospective. On one hand, reactive regulation refers to the adjustment of online action control occurring concurrently with performance: when experiencing an error (Notebaert et al., 2009) or performance difficulty (Gratton et al., 1992). For instance, reactive anticipatory mechanisms are at work when a driver slows down after passing another vehicle at high speed on a narrow road, thus reducing the risk of an accident. On the other hand, prospective regulation of online action control is not contingent on an individual’s immediate performance experience, as it involves adjustments that precede the task in question. This may consist of proactive strengthening of online action control (e.g., driving more slowly if the weather is rainy) or preempting the need to use online action control resources altogether (e.g., avoiding driving in rainy weather). Distinctions between these adaptive mechanisms and inhibitory processes have already been outlined both behaviorally (Davranche and McMorris, 2009; Wylie et al., 2009) and in terms of neural implementation (Wylie et al., 2009). Since driving is a complex and changing task, drivers are expected to be able to adapt to all conditions. However, to our knowledge, the effectiveness of these adaptive mechanisms has been less studied as a potential factor explaining driving performance, even though some of these markers can be extracted from the data collected in interference tasks. For instance, the Gratton effect (also known as the congruency sequence effect, Notebaert et al., 2009) reflects an increase in the mobilization of inhibitory processes following exposure to a conflicting situation. A higher Gratton effect in an individual is thus a marker of more robust reactive adaptive mechanisms. If these mechanisms are critically involved in driving, indices such as the Gratton effect should be predictors of safe driving. Furthermore, since the necessity to adapt is mainly present in challenging driving conditions, individuals with limited reactive adaptive mechanisms can be expected to avoid certain challenging driving scenarios.

While good cognitive functioning is a key requirement for appropriate decision-making while driving, it’s essential to recognize that individual variations in driving behaviors can also be attributed to personality traits. Particularly, previous studies have shown that self-reported assessments of risk-taking tendencies and impulsivity-related personality traits were able to predict unsafe driving (Begg and Langley, 2004; Gulliver and Begg, 2007; Curran et al., 2010; Constantinou et al., 2011). However, self-reporting methods were criticized for two main reasons. Firstly, they presuppose that participants can accurately report their behaviors, a presumption that may not always hold true (Ladouceur et al., 2000). Secondly, self-reports can be influenced by social desirability biases as participants might be hesitant to disclose risky behaviors due to apprehensions that it could result in adverse outcomes, leading them to withhold complete honesty (Lejuez et al., 2002). As a response to these limitations, some studies started to use performance-based measures of risk-taking tendency: the index obtained in these experimental tasks successfully predicted risky driving (Vaca et al., 2013; Le Bas et al., 2015), traffic offense (Lev et al., 2008; Cheng et al., 2012), and higher mean and maximum speed in simulators (Graefe and Schultheis, 2013). Nonetheless, the direct connection between behavioral measures of risk-taking as a personality trait and real-world on-road driving performance seems to have received less attention.

The present study aimed to determine whether specific inhibition-related cognitive processes, reactive adaptive mechanisms, and risk-taking tendencies were able to predict driving performance and habits among the general population. Licensed drivers of all ages completed an on-road driving performance assessment, along with a self-reported checklist of difficult driving situations usually avoided (hereafter called strategic compensation). In addition, they performed a battery of tests assessing risk-taking tendency and executive functioning, including the two different forms of inhibition (i.e., response inhibition and interference control) and the effectiveness of reactive adaptive mechanisms (i.e., the Gratton effect). We hypothesized that (a) poor driving performance would be associated with high avoidance of difficult driving situations, (b) low cognitive functioning (i.e., low inhibitory capacities and poor reactive adaptive mechanisms) would imply poorer driving performance; (c) high involvement of reactive adaptive mechanisms would lead to less avoidance of difficult driving situations and (d) high risk-taking tendency would predict poor on-road driving performance.

## 2 Materials and methods

### 2.1 Participants and materials

A total of 670 participants (336 females), aged 18 to 90 years old (*M* = 38.5, *SD* = 16.2), were recruited at 47 testing centers in France from May 2018 to September 2019. These participants underwent an evaluation that included a battery of experimental tasks and an on-road driving session with professional instructors. The battery included tests not discussed in this paper as they concerned different research aims. All task was computerized, and participants responded using a response box with buttons on top of two joysticks. The battery, which typically took about 1 h to complete, was followed by a 30-min on-road session with a driving instructor. All participants held valid driver’s license. Ethical approval for the current study was obtained from the ethics committee of the University of Lille (2017–9–S55).

### 2.2 Psychological tasks

#### 2.2.1 Simon task

The Simon task is a forced dual-choice reaction time (RT) task in which a stimulus is presented on the right or the left side of a screen (Simon, 1990). In this specific experiment, the stimulus was either a square or a circle. Participants were instructed to respond as fast and accurately as possible according to both the shape of the stimulus and the stimulus–response mapping: square - right finger press; circle - left finger press. During the task, half of the trials were congruent, meaning that the stimulus location matched the expected response, while the other half were incongruent where the stimulus location did not align with the expected response. Each trial was organized as follows: a white fixation cross first appeared in the center of a black screen for 300 ms, after which the stimulus was presented. The stimulus remained on the screen until the participant responded, with a maximum duration of 1,500 ms if no response was detected (i.e., omission). Each trial was followed by a 500 ms black screen inter-trial interval (ITI). A brief training phase of 20 trials was implemented to familiarize participants with the task instructions and the response device. During this training phase, feedback on performance was provided for 500 ms at the end of each trial. Participants performed two blocks of 129 trials each. The entire task lasted approximately 8 min.

After excluding the first trial from each block, mean RTs (ms) and error rates (%) were measured. The interference effect, calculated by subtracting the mean RTs on incongruent and congruent correct trials, represented the interference control index. Lastly, the Gratton effect, defined as the mean difference between the interference effect observed after an incongruent trial and the interference effect observed after a congruent trial, was calculated to assess reactive adaptive mechanisms (Gratton et al., 1992).

#### 2.2.2 Stop signal task

The Stop Signal task is a forced dual-choice RT task in which the engaged response must occasionally be stopped when a stop signal occurs (Logan et al., 1984). In the present case, a white arrow, pointing either left or right, was displayed at the center of the screen. In Go trials (75%), participants were required to respond as quickly as possible according to the direction of the arrow (Go signal). In Stop trials (25%), a Stop signal appeared shortly after the presentation of the Go signal, prompting participants to withhold their response by inhibiting their engaged motor command.

The Go and Stop trials started with the presentation of a white fixation cross for 300 ms before the Go signal (i.e., the arrow) and ended with a 500 ms black screen ITI after the participant’s response or a 1,500 ms delay in the absence of response. In Stop trials, the time delay between the Go and the Stop signal was initially set to 200 ms and then incrementally adjusted in 50 ms steps according to failed (−50 ms) or successful (+50 ms) stopping responses. A short training phase of 20 trials was implemented to familiarize participants with the task instructions and the response device. During training, feedback on performance was provided for 500 ms at the end of each trial. Participants performed two blocks of 129 trials. The task lasted approximately 8 min.

The Stop Signal Reaction Time (SSRT) represents the time needed for a participant to inhibit the engaged motor command after the appearance of the stop signal. A longer SSRT thus indicates weaker response inhibition performance. This index can be optimally calculated using the integration method detailed in the consensus guide of Verbruggen et al. (2019).

#### 2.2.3 Balloon analog risk task

In the Balloon Analog Risk task (BART), participants were asked to reach a goal score by virtually pumping a series of 30 simulated balloons using a button press on the joystick with their dominant hand. Pumping a balloon earned them points, but each balloon had a predefined maximum inflation time unknown to the participants. If participants exceeded this time, the balloon exploded, and they lost all cumulated points. Therefore, participants had to decide when to stop pumping to save the accumulated points.

The predefined time before explosion for a balloon ranged between 7 and 14 s (*M* = 10.0 s, *SD* = 1.8 s). Each 500 ms of pumping gave one potential point for all balloons. After pretesting, a goal score of 400 points was fixed to encourage participants to take some risks. The mean pumping time of balloons that did not explode was interpreted as the risk-taking tendency index, where a longer pumping time indicated higher risk-taking (Lejuez et al., 2002).

### 2.3 Driving behavior assessment

Participants performed a 30-min on-road session with a professional driving instructor. The instructor filled out a French version of the Test Ride for Investigating Practical Fitness to Drive (TRIP) (De Raedt and Ponjaert-Kristoffersen, 2001; Ranchet et al., 2013). This instrument consists of an evaluation grid of 62 items that assess the participant’s driving ability on multiple components of the driving task (e.g., lane positioning, car following, speed, visual exploration, traffic signs, overtaking, anticipatory behavior, communication, turning left, merging on another lane, and mechanical operations). Each item is rated on a four-point scale, ranging from 1 (“insufficient”) to 4 (“good”).

A fixed itinerary was created for each testing center to encounter as many traffic situations as evaluated in the TRIP. Despite that, some participants did not encounter specific situations. Consequently, six items concerning these situations were excluded from the analyses: two regarding overtaking, two related to communication with others, and two concerning rural roads driving. The overall TRIP score was standardized on a scale of 100 using the cross-multiplication method. A TRIP score below 58 is assumed to indicate poor driving performance (Ranchet, 2011).

In addition to the on-road session, participants’ level of strategic compensation was assessed using a self-report questionnaire consisting of 16 items representing a list of difficult driving conditions (e.g., high traffic, night driving). Participants were instructed to check each condition they typically avoid while driving (Ranchet et al., 2013). The strategic compensation score ranged from 0 to 16, with one point attributed for each driving condition avoided. This score served as an index of the preemptive form of prospective anticipatory action regulation.

### 2.4 Inclusion criteria

A set of inclusion criteria was applied for both the Simon task and the Stop signal task to limit our sample to participants who properly respected the instructions. For the Simon task, participants with an error rate below 15% and an omission rate below 10% were kept for the analyses. For the Stop Signal task, following the recommendations of Verbruggen et al. (2019), the inclusion criteria for the analyses were as follows: a proportion of successfully stopped trials between 25 and 75%, a mean failed stop trial RT strictly inferior to the mean go trial RT, a go trial response rate of at least 60%, a go error rate below 10%, and an SSRT above 0 ms. According to these criteria, statistical analyses were performed on a sample of 589 participants (296 females). The participants’ ages ranged from 18 to 90 years old (*M* = 37.7, *SD* = 16.2).

### 2.5 Statistical analysis

A mixed-effect model was fitted using the *lme4* (Bates et al., 2015) and *lmertest* (Kuznetsova et al., 2017) R packages to evaluate the impact of the performances at each of the psychological tests on the TRIP score. Age was also added as a fixed effect to replicate previous findings showing a decline in the fitness-to-drive with normal aging (Adrian et al., 2019). The driving instructor was set as a random intercept effect to account for the inter-instructor variability. A multiple regression analysis was applied to examine the effect of the tasks’ performance variables and age on the strategic compensation score.

## 3 Results

### 3.1 Preliminary data analysis

Results of descriptive statistics for all psychological tasks and the driving measures are presented in Table 1. Since most variables were not normally distributed, correlation analyses were performed using Kendall’s tau. The correlation matrix of all variables is displayed in Supplementary material. Age correlated positively, although weakly, with the SSRT from the Stop Signal task, *r_τ_* = 0.16, *p < 0*.001, confirming previous results showing a decline in response inhibition abilities with age. The Gratton effect and the interference effect from the Simon task also weakly correlated with age, *r_τ_* = 0.18, *p < 0*.001, and *r_τ_* = 0.08, *p* = 0.006, respectively. Older participants were more sensitive to the interference but also exhibited a higher involvement of reactive adaptive mechanisms. Finally, the mean pumping time from the BART weakly correlated with age, *r_τ_* = −0.08, *p* = 0.005, indicating that risk-taking decreased with increasing age.

**Table 1:** Descriptive statistics for all dependent variables from the experimental tasks and both driving scores.

Tasks	Variables of interest	Mean	SD	1*^st^* Qrt.	Median	3*^rd^* Qrt.
Simon task	Mean RT (ms)	535.64	74.64	482.44	528.36	579.10
	Error rate (%)	3.07	2.35	1.17	2.33	4.26
	Omission rate (%)	0.32	0.94	0	0	0.39
	Interference effect (ms)	24.59	22.96	11.06	23.53	38.03
	Gratton effect (ms)	104.40	46.13	72.08	104.33	133.32
	Mean Go RT (ms)	664.47	183.73	524.58	616.07	775.33
	Error rate (%)	1.16	1.54	0	0.52	1.55
	Omission rate (%)	1.87	4.10	0	0	1.55
Stop Signal task	SSRT (ms)	243.86	67.02	206	244.77	280.85
BART	Mean pumping time (s)	7.67	0.78	7.35	7.78	8.12
	Number of exploded balloon (/30)	5.20	3.16	3	5	7
Driving	TRIP score	85.53	11.15	77.93	86.85	95.77
	Strategic compensation score	1.89	2.02	0	1	3

RT, reaction time; SD, standard deviation; Qrt., Quartile.

**Figure 1:**
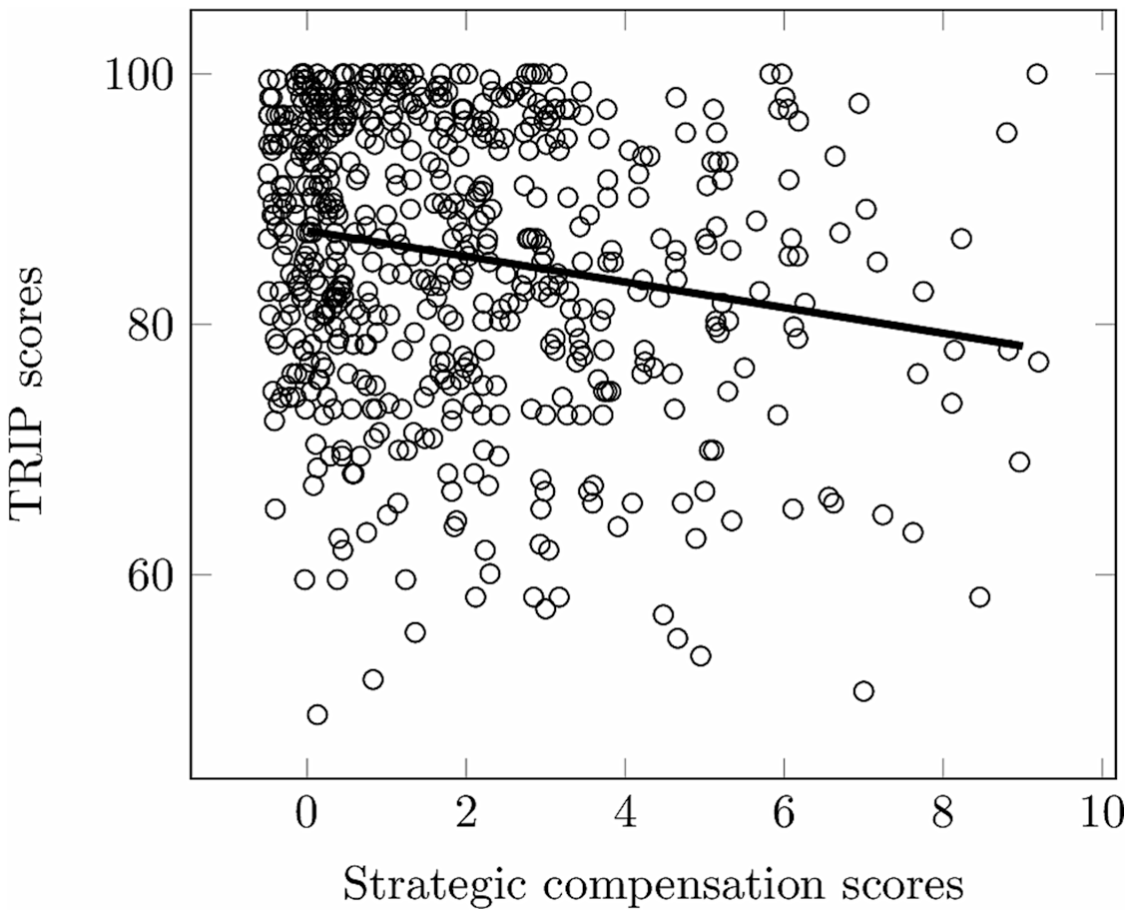
Correlation between the TRIP score and the strategic compensation score. Each jittered point represents one participant. The TRIP score was given by the driving instructor after the 30-min on-road assessment. The strategic compensation was measured on a 16-item self-reported questionnaire.

Additionally, the on-road TRIP score, assessed by driving instructors, negatively correlated with the self-reported strategic compensation, *r_τ_* = −0.12, *p < 0*.001 (Figure 1). This indicates that better driving performance was associated with lower avoidance of difficult driving situations.

### 3.2 On-road driving performance

The TRIP score ranged from 48.83 to 100, with eight individuals scoring below the threshold for poor driving performance (Ranchet, 2011). The TRIP score was analyzed in relation to measures from the battery of tests assessing risk-taking tendency, reactive adaptive mechanisms, and two different forms of inhibition: response inhibition and interference control (i.e., fixed effects). A Kruskal-Wallis test revealed that the median TRIP score varied among instructors, *H*(15) = 237, *p* < 0.001. Their identifier was therefore set in the model as a random effect. The final mixed model was TRIP ~ DV + AGE + (1|INSTRUCTOR), with DV referring to the variables displayed in Table 2.

**Table 2:** Driving performance scores model *t*-statistics of predictors.

Predictors	TRIP score			Strategic compensation score		
	*Beta*	*t*	*p*	*Beta*	*t*	*p*
Mean RT (ms)	−0.017	−2.75	0.006**	0.002	1.64	0.101
Error rate (%)	0.014	0.08	0.935	0.018	0.47	0.639
Interference effect (ms)	−0.017	−1.01	0.309	0.001	0.40	0.690
Gratton effect (ms)	0.011	1.17	0.244	−0.006	−2.99	0.003**
SSRT (ms)	−0.006	−0.90	0.366	0.001	0.86	0.393
Average pumping time (s)	1.313	2.65	0.008**	−0.170	−1.57	0.117
Age	−0.020	−0.68	0.495	0.013	2.17	0.030*
*R^2^*	0.036 [0.024, 0.079]			0.030 [0.001, 0.051]		

Left column: *t*-statistic of each predictor (variables from the experimental tasks and age) for the mixed model applied to the TRIP score, when the inter-instructor variability is accounted for. Right column: *t*-statistic of the same predictors for the regression analysis applied to the strategic compensation score. ^*^p < 0.050, ^**^p < 0.01. R^2^ was expressed as marginal R^2^ (i.e., part of the variance explained by the fixed effects only; Nakagawa and Schielzeth, 2013) for the linear mixed model (TRIP score) and as adjusted R^2^ for the multiple regression analysis (Strategic compensation score). Bold values refer to significant contributions.

Table 2 displays the *t*-statistics for each fixed effect in the model (i.e., test measures from the experimental tasks and age). Interestingly, neither the interference effect nor the SSRT predicted the TRIP score. Age also failed to predict the TRIP score. However, individuals with higher mean RT in the Simon task showed a reduced TRIP score. Contrary to our expectations, higher mean pumping time in the BART, revealing a higher risk-taking tendency, predicted a higher TRIP score.

### 3.3 Strategic compensation

Given that the answers to the strategic compensation questionnaire were not influenced by the driving instructors, a regression analysis was sufficient to test the effect of the experimental measures and age on the strategic compensation score. Table 2 provides an overview of the *t*-statistics for each model parameter. Notably, the inhibition indices, i.e., interference effect and SSRT, did not predict the strategic compensation score. In addition, the strategic compensation score increased with age but decreased in individuals showing a higher Gratton effect in the Simon task.

## 4 Discussion

### 4.1 Summary of main results

Our study aimed to determine whether specific inhibition-related cognitive processes, involvement of reactive adaptive mechanisms, and risk-taking tendencies were able to predict driving performance and habits among the general population. We sought to identify which of these variables contributed the most to explaining driving performance and habits assessed by a 30-min on-road test and a questionnaire, respectively. We expected that poorer inhibition capacities, weaker reactive adaptive mechanisms, and higher risk-taking would lead to poorer on-road driving performance. Unexpectedly, our findings indicated that a high risk-taking tendency predicted better on-road driving performance, while both types of inhibition and reactive adaptive mechanisms showed no significant effect. However, we confirmed that higher self-reported avoidance of difficult driving situations is associated with poorer driving performance. Moreover, we hypothesized that high involvement of reactive adaptive mechanisms would lead to less avoidance of difficult driving situations, as assessed by the strategic compensation questionnaire. This hypothesis was confirmed by our findings.

### 4.2 The role of inhibition

Recent work showed that among the executive functions, inhibition seemed to be the most critical to assess on-road driving performance (Adrian et al., 2019). In their study, Adrian et al. (2019) found that inhibition fully mediated the effect of normal aging on driving performance. However, inhibition was assessed as a whole, despite the existence of multiple forms of inhibition. Thus, we sought to extend this finding by exploring the level of contribution of two distinct forms of inhibition: response inhibition and interference control. Unexpectedly, neither of them, nor age, predicted driving performance. Contrary to what is reported in Adrian et al. (2019), age seems not to be a significant predictor of driving performance and fitness-to-drive. In fact, Toepper et al. (2021) reported that older drivers with mild cognitive impairment were more unfit to drive than healthy older drives. In our sample, the older participants did not have an established cognitive diagnosis, which may explain the observed weak correlations between inhibition capacities and age. Another possible explanation for the absence of results could be the duration of the on-road session. In our study, the session lasted approximately 30 min, while in Adrian et al. (2019), the TRIP evaluation involved 150 min of driving. Since having good inhibition capacities is likely critical only in episodic scenarios, 30 min might be too short to experience these scenarios and, consequently, to observe the impact of inhibition (and hence, age) on driving performance. Additionally, the TRIP evaluation grid remains general when it comes to different dimensions of driving; in fact, no item directly tackles inhibitory behaviors. In contrast, the fact that we observed an increased strategic compensation among older persons shows that individuals become less confident in their driving skills as they grow older. In the current study, we replicated previous findings that avoidance behaviors are associated with low driving skills and less fitness-to-drive in older drivers (Schulz et al., 2020; Toepper et al., 2021), but in a broader age sample. Assuming that the decrease in confidence is due to a genuine decline in performance, then it further suggests that the TRIP assessment may not be sensitive to all aspects of driving performance. As individuals age, their inhibition capacities, especially response inhibition, tend to deteriorate, as our results, and those of other studies, suggest (Hsieh et al., 2016; Ferguson et al., 2021). Therefore, it is highly likely that this decline contributes to why older individuals tend to avoid more challenging driving situations.

### 4.3 The importance of adaptive mechanisms

Adaptive mechanisms lead to anticipatory contextual adjustments of cognitive processes, including inhibition. A subset of these mechanisms is called reactive, which involves adjusting future behavior in response to experienced errors or performance difficulty. To the best of our knowledge, the present study is the first to explore the impact of the involvement of these reactive adaptations on driving performance. As mentioned in the previous subsection, the relatively short duration of the on-road session made it unlikely to encounter particularly challenging situations. The necessity to adapt was minimal, it was therefore not surprising that we observed no impact of reactive adaptive mechanisms on on-road performance in this context. Nevertheless, the present findings indicate that reactive adaptation is associated with less strategical avoidance of difficult driving situations. This result confirms our hypothesis that individuals who are more able to reactively adapt their behaviors are likely to possess greater confidence in their ability to cope with difficult situations. Strengthening these adaptive mechanisms, especially among older populations, could offer a novel approach to restoring confidence in driving skills.

### 4.4 The relationship between risk-taking tendency and driving

Probably the most surprising result of this study is the effect of risk-taking tendency on driving performance. Contrary to our hypothesis, which was based on previous studies, a higher risk-taking tendency was found to predict a better fitness-to-drive in our sample. Strangely, even though the TRIP evaluation grid includes multiple items directly relevant to safety (e.g., maintaining an appropriate speed and an appropriate distance to other vehicles) that should punish risky behaviors, individuals with higher risk-taking tendencies still showed better scores. This does not necessarily mean that risk-takers are safer drivers. A risk-taking tendency can be considered as a personality trait that biases decision-making toward riskier choices. In a driving context, where risk-taking can lead to accidents, this trait may appear maladaptive. In the present context, the presence of the driving instructor in the car may have counterbalanced this decision bias by pressing participants to adopt a safer attitude than usual. Among participants with a low risk-taking tendency, this increased caution induced by the presence of the driving instructor might have become “unsafe,” or at least maladjusted to the context. Previous studies have observed impaired driving performances due to being overly cautious in investigations of stereotypes among older drivers (Joanisse et al., 2012; Lambert et al., 2016). While we presently cannot verify if non-risk-takers showed signs of overly cautious driving, our findings do suggest an overall more complex relationship between risk-taking tendency and driving performance in an ecological context of on-road driving.

### 4.5 General conclusion

The current study has highlighted the limitations of using inhibition tests to predict on-road performance in the general population. As long as executive functions are operational, personality factors, such as risk-taking tendencies and perhaps the level of self-confidence, appear to be more effective criteria for assessing fitness-to-drive.

## Data availability statement

The datasets presented in this article are not readily available because they were collected and are owned by the private company ECCA Conduite. There are thus restrictions on the availability of these data, which were used under a specific contract for the current study and are not publicly available. The data used in this study can be made available by the SCALab, upon receipt of permission from the ECCA Conduite company. Requests to access the datasets should be directed to contact-scalab@univ-lille.fr and contact@ecca-conduite.eu.

## Ethics statement

The studies involving humans were approved by the ethics committee of the University of Lille. The studies were conducted in accordance with the local legislation and institutional requirements. The participants provided their written informed consent to participate in this study.

## Author contributions

FG, YD-T, AD, ID-EG, and CR: conceptualization. PD, YD-T, and CR: data curation. PD, FG, QV, TD, and CR: formal analysis. YD-T, AD, ID-EG, and CR: funding acquisition. AD and ID-EG: data Curation, investigation and resources. PD, FG, YD-T, QV, TD, and CR: methodology. YD-T, TD, AD, ID-EG, and CR: project administration. YD-T and CR: supervision. PD, FG, and CR: visualization. PD: writing – original draft. PD, FG, YD-T, and CR: writing – review & editing. All authors contributed to the article and approved the submitted version.

## Footnotes

^1^Since reading acts as a distractor to ignore, the Stroop task is also sometimes considered as an interference control task (Nigg, 2000).
