# Supplementary material for: Investigating risk-taking and executive functioning as predictors of driving performances and habits: a large-scale population study with on-road evaluation
Source: Front Psychol. 2023 Nov 27;14:1252164. doi: 10.3389/fpsyg.2023.1252164 (PMC10711047; doi:10.3389/fpsyg.2023.1252164)
Supplement: Supplementary file 1 [file Data_Sheet_1.docx]

Supplementary Material

|  | **Table A. Correlation matrix of all variables in the study** | | | | | | | | | | | | | | | | | | | | | | | | | | | | | |  |  |  |  |  |  |
| --- | --- | --- | --- | --- | --- | --- | --- | --- | --- | --- | --- | --- | --- | --- | --- | --- | --- | --- | --- | --- | --- | --- | --- | --- | --- | --- | --- | --- | --- | --- | --- | --- | --- | --- | --- | --- |
|  | **Variables** |  | | | **1.** | | **2.** | | | **3.** | | | **4.** | | | | | **5.** | | | **6.** | | | **7.** | | | **8.** | | | **9.** | | | **10.** | |  |  |
| Simon task | 1. Mean RT (ms) |  | Kendall's Tau |  | | — | |  |  | |  |  | |  |  |  |  | |  |  | |  |  | |  |  | |  |  | | |  | |  | |  |
|  |  |  | p-value |  | | — | |  |  | |  |  | |  |  |  |  | |  |  | |  |  | |  |  | |  |  | | |  | |  | |  |
|  | 2. Error rate (%) |  | Kendall's Tau |  | | -0.164 | |  | — | |  |  | |  |  |  |  | |  |  | |  |  | |  |  | |  |  | | |  | |  | |  |
|  |  |  | p-value |  | | **< .001** | |  | — | |  |  | |  |  |  |  | |  |  | |  |  | |  |  | |  |  | | |  | |  | |  |
|  | 3. Interference effect (ms) |  | Kendall's Tau |  | | 0.045 | |  | 0.030 | |  | — | |  |  |  |  | |  |  | |  |  | |  |  | |  |  | | |  | |  | |  |
|  |  |  | p-value |  | | 0.105 | |  | 0.279 | |  | — | |  |  |  |  | |  |  | |  |  | |  |  | |  |  | | |  | |  | |  |
|  | 4. Gratton effect (ms) |  | Kendall's Tau |  | | 0.261 | |  | 0.100 | |  | 0.033 | |  | — |  |  | |  |  | |  |  | |  |  | |  |  | | |  | |  | |  |
|  |  |  | p-value |  | | **< .001** | |  | **< .001** | |  | 0.231 | |  | — |  |  | |  |  | |  |  | |  |  | |  |  | | |  | |  | |  |
| Stop Signal task | 5. Mean Go RT (ms) |  | Kendall's Tau |  | | 0.236 | |  | -0.175 | |  | -0.033 | |  | -0.005 |  | — | |  |  | |  |  | |  |  | |  |  | | |  | |  | |  |
|  |  |  | p-value |  | | **< .001** | |  | **< .001** | |  | 0.235 | |  | 0.851 |  | — | |  |  | |  |  | |  |  | |  |  | | |  | |  | |  |
|  | 6. Error rate (%) |  | Kendall's Tau |  | | 0.014 | |  | 0.224 | |  | 0.074 | |  | 0.099 |  | -0.349 | |  | — | |  |  | |  |  | |  |  | | |  | |  | |  |
|  |  |  | p-value |  | | 0.648 | |  | **< .001** | |  | **0.014** | |  | **< .001** |  | **< .001** | |  | — | |  |  | |  |  | |  |  | | |  | |  | |  |
|  | 7. SSRT (ms) |  | Kendall's Tau |  | | 0.188 | |  | 0.010 | |  | 0.082 | |  | 0.082 |  | -0.123 | |  | 0.142 | |  | — | |  |  | |  |  | | |  | |  | |  |
|  |  |  | p-value |  | | **< .001** | |  | 0.725 | |  | **0.003** | |  | **0.003** |  | **< .001** | |  | **< .001** | |  | — | |  |  | |  |  | | |  | |  | |  |
| BART | 8. Mean pumping time (s) |  | Kendall's Tau |  | | -0.086 | |  | 0.044 | |  | -0.024 | |  | -0.069 |  | -0.069 | |  | -0.025 | |  | -0.059 | |  | — | |  |  | | |  | |  | |  |
|  |  |  | p-value |  | | **0.002** | |  | 0.116 | |  | 0.390 | |  | **0.012** |  | **0.012** | |  | 0.406 | |  | **0.034** | |  | — | |  |  | | |  | |  | |  |
|  | 9. Number of exploded balloons ( /30) |  | Kendall's Tau |  | | -0.044 | |  | 0.069 | |  | 0.020 | |  | 0.029 |  | -0.061 | |  | 0.022 | |  | -0.029 | |  | 0.569 | |  | — | | |  | |  | |  |
|  |  |  | p-value |  | | 0.130 | |  | **0.019** | |  | 0.490 | |  | 0.321 |  | **0.035** | |  | 0.484 | |  | 0.315 | |  | **< .001** | |  | — | | |  | |  | |  |
|  | 10. Age |  | Kendall's Tau |  | | 0.318 | |  | -0.182 | |  | 0.076 | |  | 0.184 |  | 0.148 | |  | 0.018 | |  | 0.160 | |  | -0.078 | |  | -0.075 | | |  | | — | |  |
|  |  |  | p-value |  | | **< .001** | |  | **< .001** | |  | **0.006** | |  | **< .001** |  | **< .001** | |  | 0.546 | |  | **< .001** | |  | **0.005** | |  | **0.010** | | |  | | — | |  |

**Split-half reliability indices of the main measures of the Simon and the Stop Signal tasks.**

- Interference effect: *r* = 0.47, 95% CI [0.40, 0.53]
- Gratton effect: *r* = 0.46, 95% CI [0.39, 0.52]
- SSRT: *r* = 0.95, 95% CI [0.94, 0.96]

The reliability measures for both the interference effect and the SSRT are consistent with recent work (Thunberg et al., 2023; Hedge et al., 2022). In the case of the Gratton effect, we did not find studies that provided a comparable index of comparison.

**Instructors’ internal consistency measures.**

Across monitors, the Cronbach’s alpha was on average 0.65 (*SD* = 0.33), and ranged from -0.06 and 0.93. Internal consistency was lower than the commonly accepted satisfactory threshold of 0.70 (Taber, 2018) for five instructors. We thus tested the robustness of our main findings by fitting again our linear mixed model without them. None of the findings changed qualitatively (see the Table below), indicating that our results were not affected by the lack of internal consistency of some of the driving instructors.

| **Predictors** | **TRIP score** | | |
| --- | --- | --- | --- |
|  | ***β*** | ***t*** | ***p*** |
| Mean RT (ms) | -0.026 | -2.56 | **.011*** |
| Error rate (%) | 0.31 | 1.04 | .299 |
| Interference effect (ms) | -0.016 | -0.54 | .589 |
| Gratton effect (ms) | 0.004 | 0.23 | .819 |
| SSRT (ms) | -0.004 | -0.43 | .671 |
| Average pumping time (s) | 1.867 | 2.39 | **.018*** |
| Age | -0.038 | -0.691 | .490 |
| R² [95% CI] | 0.071 [0.039, 0.155] | | |

*Note*. *: *p* < .050, **: *p* < .01. R² is expressed as marginal R² (i.e., part of the variance explained by the fixed effects only; Nakagawa & Schielzeth, 2013).

**Mixed model with interaction fixed effects between psychological measures and Age.**

To test the hypothesis that measures of cognitive control become diagnostic only at a certain age, and not before, we fitted an alternative linear mixed model with interactions between Age and each predictor as fixed effects, resulting in the following formula:

*TRIP ~ Mean RT + Error rate + Interference effect + Gratton effect + SSRT + Average pumping time + Age + Age:Mean RT + Age:Error rate + Age:Interference effect + Age:Gratton effect + Age:SSRT + Age:Average pumping time + (1|Instructor).*

Adding the interactions did not substantially improve the goodness of fit of the model compared to the original model without interaction effects. Specifically, the Akaike Information Criterion (AIC) for the model without interaction was 4301.8, while the AIC for the model with age interactions was 4310.8. This result suggests that measures of cognitive control do not become (more) diagnostic at a certain age.
